# An Effective Image Denoising Method for UAV Images via Improved Generative Adversarial Networks

**DOI:** 10.3390/s18071985

**Published:** 2018-06-21

**Authors:** Ruihua Wang, Xiongwu Xiao, Bingxuan Guo, Qianqing Qin, Ruizhi Chen

**Affiliations:** 1 State Key Laboratory of Information Engineering in Surveying, Mapping and Remote Sensing, Wuhan University, Wuhan 430079, China; auspicioushua@sina.com (R.W.); 00201550@whu.edu.cn (B.G.); ruizhi.chen@whu.edu.cn (R.C.); 2 Department of Mathematics, University of California, Irvine, CA 92697, USA

**Keywords:** UAV images, image denoising, generative adversarial networks, perceptual reconstruction loss

## Abstract

Unmanned aerial vehicles (UAVs) are an inexpensive platform for collecting remote sensing images, but UAV images suffer from a content loss problem caused by noise. In order to solve the noise problem of UAV images, we propose a new methods to denoise UAV images. This paper introduces a novel deep neural network method based on generative adversarial learning to trace the mapping relationship between noisy and clean images. In our approach, perceptual reconstruction loss is used to establish a loss equation that continuously optimizes a min-max game theoretic model to obtain better UAV image denoising results. The generated denoised images by the proposed method enjoy clearer ground objects edges and more detailed textures of ground objects. In addition to the traditional comparison method, denoised UAV images and corresponding original clean UAV images were employed to perform image matching based on local features. At the same time, the classification experiment on the denoised images was also conducted to compare the denoising results of UAV images with others. The proposed method had achieved better results in these comparison experiments.

## 1. Introduction

As a rapid evolution technology, the increased availability of unmanned aerial vehicles (UAVs) has drawn attention for their capability to generate ultra-high spatial resolution images. UAV however is a low-altitude remote sensing platform, which is affected by lightning, ground electromagnetic waves, illumination change, and mechanical noise from the UAV itself. These factors are sources of noise in UAV images, therefore, it is especially important to study how to remove noise from UAV images. 

In recent years, there is a growing body of research on noise removal in remote sensing images. Liu et al. [1] used an auxiliary noise-free image as a prior, proposing a denoising method for remote sensing images based on partial differential equations. Rajapriyadharshini et al. [2] split the noisy images into several disjoint local regions and clustered the noisy images into several disjoint local regions to denoise SAR images. Bhosale et al. [3] designed wavelet filter to restore the remote sensing images and explored the effects of noise. Wang et al. [4] proposed high-order balanced multi-band multiwavelet packet transforms to denoise remote sensing images and claimed that an appropriate band number can improve the denoising performance. Xu et al. [5] provided a method based on blockwise nonlocal means algorithm to denoise repetitive image patches in remote sensing images. Penna et al. [6] utilized non local means with the stochastic distances to denoise SAR images. Kim et al. [7] adopted background registration processing and robust principle component analysis, proposing a method of noise filtering of LWIR/MWIR Imaging sensors. 

Some researchers studied the denoising of remote sensing images based on sparse expression. Chang et al. [8] combined unidirectional total variation and sparse representation to learn a dictionary trained to fit the input data to remove random noises from remote sensing images. Cerra et al. [9] reduced the weight of sparse unmixing problem proposing a denoising method based on sparse reconstruction of simulated EnMAP data. Xu et al. [10] used the nonlocal sparse model and the iterative regularization technique to denoise SAR images.

Nowadays, deep learning is breeding new ideas and convolutional neural networks (CNNs) have become recognized as an efficient method that automatically learns deep-level feature representations from images. Denoising algorithms for natural images, based on deep learning are an emerging trend. Jain et al. [11] synthesized training samples from specific noise models and used convolutional networks as unsupervised learning procedure and image processing architecture. Xie et al. [12] put forward a method of image denoising that combined sparse representation and deep neural networks pre-trained by denoising auto-encoders. Burger et al. [13] introduced a plain multi-layer perceptron of mapping from a noisy image to a noise-free image. Wu et al. [14] used rectified linear function instead of sigmoid function as the hidden layer activation function of deep neural networks to achieve image denoising. Xu et al. [15] provided a method of using deep convolutional neural networks as reliable a support for robust deconvolution against artifacts for image restoration. Li et al. [16] combined sparse coding and auto-encoder to achieve image denoising. Mao et al. [17] put forward networks with multiple layers of convolution and deconvolution operators to learned end-to-end mappings from a denoised image to a clean image. 

Many practical remote sensing applications require clear textures for ground objects in remote sensing images. Because of the wide range of remote sensing images, remote sensing image denoising must consider the overall spatial distribution of ground objects, which is different from the natural images. It is easier to extract deep texture features in remote sensing images from the deeper neural network structure. Nevertheless, the deeper networks are often more complicated to train because of the internal covariate shift. Batch normalization can address this problem due to the fact that it back propagates the gradients through the normalization parameters and preserves the representation ability of the networks [18,19,20]. Residual learning framework solves the gradient vanishing of deeper neural networks because it explicitly lets each few stacked layers fit a residual mapping instead of expecting these layers directly fit a desired underlying mapping [21,22,23,24]. Visual Geometry Group networks (VGGs) are beneficial for the deep-level representation of images [25,26,27], which is of great help for modeling mappings of highly complex ground features. Johnson et al. [27] utilized pre-trained VGG nets to extract high-level features by optimizing the perceptual loss function. Kiasari et al. [28] used perceptual loss to alleviate the problem of blurry image generation. Generative Adversarial Networks (GAN) with constantly improving optimization strategies have been applied widely to image generation and classification problems [20,22,29,30]. Dosovitskiy et al. [31] combined Euclidean distances with GAN to calculate the distances between image features extracted.

On the basis of distinctive demand for texture features from UAV images and combining with the continuous development of deep learning methodology, we propose a method based on generative adversarial networks to obtain clearer feature textures for denoised UAV images. In this paper, pretrained VGG networks are employed to extract deep-level complex features of UAV images and a perceptual reconstruction loss is combined with the pixel-based Euclidean loss for constant optimizations of the game theoretic min-max model to generate fake clean UAV images using powerful generation capability of GAN. In order to make the networks structure deeper and sturdier, the batch-normalization is used to regularize training data and multiple residual blocks are utilized to build the networks.

## 2. Methods

### 2.1. Deep Architecture

GAN can effectively learn the distributions of clean and noisy UAV images, using clean UAV images to denoise UAV images. The proposed neural network based on GAN directly learns the mapping between clean UAV images and noisy UAV images, based on the work of Goodfellow et al. [29], who generated realistic data by effectively learning the distributions of a training data set. They adopted a min-max adversarial game theoretic optimization framework to train a generative model G and discriminative model D simultaneously. GAN continuously train a model G so that the probability distributions of the images generated by G are indistinguishable from real images, thus fooling model D. According to this idea, we consider the UAV image image-denoising problem as a generative adversarial problem, aiming to learn a mapping directly from clean UAV images to noisy UAV images by constructing a GAN-based deep networks called Denoise UAV image Generative Adversarial Network (DUGAN). In order to learn a generator that can fool a discriminative model (D) so it can distinguish clean UAV images from generated denoised UAV images. Thus, a generative model G and a discriminative model D were designed.

#### 2.1.1. Generative Model

**Figure 1:**
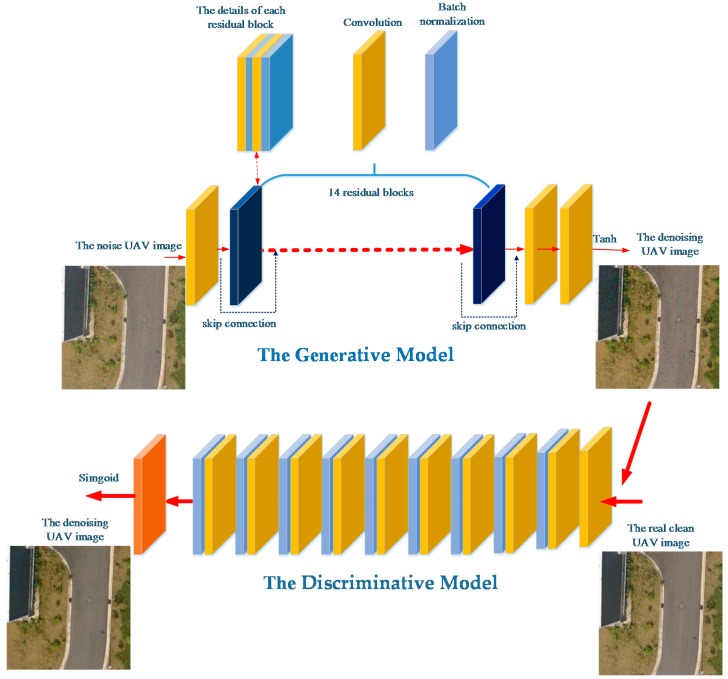
An overview of the proposed DUGAN method. The networks include generative model G and discriminative model D.

The primary aim of generative model G is to generate clean denoised UAV images from real clean UAV images. As the original GAN model is unstable, artifacts in the output images are synthesized by generative model G, which do not meet ground-object texture requirements of UAV image applications. Therefore, the critical is to design a deeper structure to generate denoised UAV images. We constructed a generative model G containing 14 residual blocks (Figure 1) to train the deeper networks in the G model, efficiently. 

The gradient can easily vanish in the process of back propagation while training the deeper networks, which may result in the losses of image details and textures. The residual networks make a reference to each layer’s input and learn a residual function rather than learn some functions that do not have references. This residual function is easier to optimize to solve the gradient vanishing problem structurally, at the level of the deeper networks, but greatly increases the number of network layers In the G model. Each residual block of generative model G comprises two convolutional layers and two batch normalization layers, then a skip connection is established in each residual block. Skip connection back-propagates the gradient to deeper layers and can pass gradients through multiple residual blocks smoothly, which helps recover the details in UAV images and assists the CNN to effectively denoise UAV images. 

The distributions of data will have influence on the training of deep networks. When the depth of a network increases, the overall distribution of the activation input gradually approaches the upper and lower limits of the value interval of the non-linear function of the value interval, resulting in a slow network convergence. Batch normalization forces the distributions of the input values of any neuron in each layer of the neural networks back to the standard normal distribution, so that the active input values fall into the sensitive area of the nonlinear function. Small changes can lead to large changes in the loss function, which makes the training faster and easier. In our generative model G, the activation functions of two batch normalization layers in the residual blocks are relu. In our generative model G, the activation functions of two batch normalization layers in the residual blocks are relu. In addition to these residual blocks, we add two convolutional layers to generate simulated clean UAV images using the activation function, tanh. The specific settings of each layer of the generative model are as follows:

C(r,64)→C(64)B(r)C(64)B(r)SC→…12…→C(64)B(r)C(64)B(r)SC→C(64)→C(t,3)

Here, C(r,64) denotes a set of convolutional layers with 64 feature maps and activation function relu; C(64)B(r)C(64)B(r)SC represents a residual block; B(r) is a batch normalizational layer with activation function relu, and SC denotes a skip connection. There are in total, 14 residual blocks. C(t,3) represents a convolutional layer with three feature maps and activation function tanh.

#### 2.1.2. Discriminative Model

GAN continuously trains the generative model G until the generated images cannot be differentiated by the discriminative model D from these actual, real image samples. Generative model G continuously generates better images so that the distributions of the images are undifferentiated from the distributions of real images. The discriminative model D is employed to distinguish simulated clean UAV images synthesized by the generative model from the corresponding actual clean UAV images. Model D can also be regarded as a judge and guidance for the generative model G. We constructed a discriminative model D that alternately updates G and D to solve the min-max adversarial game-theoretic optimization problem (Equation (1)):

(1) $$\underset{G}{\min}\;\underset{D}{\max}\;E_{G^{CI}∼p_{g}}[\log(1-D(G^{CI}))]+E_{I^{C}∼p_{r}}[\log D(I^{C})]$$

Here, *I^C^* represents the actual real clean image and $G^{CI}$ denotes the generated clean image, $p_{r}$ is the sample distribution of the real clean image, $p_{g}$ is the sample distribution of the generated clean image. The proposed discriminative model is shown at the bottom part of Figure 1. The specific settings of each layer of the discriminative model are as follows:

C(lr,64)→C(128)BN(lr)→C(256)BN(lr)→C(512)BN(lr)→C(1024)BN(lr)→C(2048k)BN(lr)→C(128)BN(lr)→C(128)BN(lr)→C(512)BN(lr)→SC→D

Here, lr denotes the activation function leakyrelu; C(128)BN(lr) denotes a set of convolutional layers with 128 feature maps followed by batch-normalization with activation function leakyrelu, and D is the dense layer for outputting generated clean UAV images that are indistinguishable from actual real clean UAV images. The feature maps were increased from 256 to 2048, and suitable for pre-trained VGG networks [32]. 

### 2.2. Loss Function

The definition of loss function is critical for the performance of the proposed method. Some losses of image restoration are optimized at pixel-level [31,33,34] so that images are typically, overly smooth and thus lack high frequency content and have poor perceptual quality. Some researchers argued that reconstructed results would be better to optimize a perceptual loss function by minimizing perceptual differences between reconstructed images and the true ground images [27,35,36]. The quality of the image features can be improved by the perceptual reconstruction loss so that it can meet the requirements of UAV images for features acquired from ground objects textures. GAN have powerful ability of image generation by alternatively updating generative networks G and discriminative networks D. Dosovitskiy et al. [31] combined Euclidean distances with generative adversarial training to establish a loss function. The solutions of pixel loss optimization problems often result in perceptually unsatisfying solutions with overly smooth textures. Combining the perceptual reconstruction loss function with VGG networks the networks will encourage networks to enjoy feature representations of noisy images similar to those of actual clean images. Therefore, we propose a new advanced loss equation for better denoising results of UAV images. Perceptual reconstruction loss, generative adversarial loss and Euclidean loss are combined together to formulate the proposed loss function, which is as follows:

(2) $$L_{DUGAN}=xL_{pe}+yL_{ga}+L_{pi}$$

Here, $L_{pe}$ is perceptual reconstruction loss, an appropriate measure for features extracted from a pretrained VGG networs instead of low-level pixel-wise error measures; $L_{ga}$ is adversarial loss; and $L_{pi}$ is pixel loss between noisy pixel of the noisy UAV images and pixel of the clean UAV images. *x* and *y* are respectively the weights of $L_{pe}$ and $L_{ga}$. The perceptual reconstruction loss based on the relu activation layers of the pretrained 19 layer VGG networks is defined as in [26,30]. The aim is to minimize the distances between high-level features, and $L_{pe}$ defined as Equation (3):

(3) $$L_{pe}=\frac{1}{C_{i}W_{i}H_{i}}\sum_{c=1}^{C_{i}}\sum_{w=1}^{W_{i}}\sum_{h=1}^{H_{i}}\left||V(UI_{CI}{}^{c,w,h})-V(UI_{GCI}{}^{c,w,h}\right)|{|}_{2}^{2}$$

Here, $C_{i}$, $W_{i}$, $H_{i}$ represent the channels, width and height of the images respectively, and *V* represents a non-linear CNN transformation pretrained by VGG19. $UI_{GCI}$ denotes generated clean UAV image and $UI_{CI}$ denotes the corresponding actual clean UAV image.

The generative adversarial loss encourages our networks to obtain better solutions laying in the manifold of reconstructed images by trying to fool the discriminative model, which is defined based on the probability that the discriminant model considers the generated denoised UAV images to be actual, clean UAV images, as shown in Equation (4):

(4) $$L_{ga}=-\sum_{n=1}^{N}\log D(UI_{GCI})$$

Here, $D(UI_{GCI})$ is the probability that the generated images are real clean UAV images. We minimize $L_{ga}$ continuously for better denoising results of UAV images. The per-pixel Euclidean loss is defined as Equation (5):

(5) $$L_{pi}=\frac{1}{CWH}\sum_{c=1}^{C}\sum_{w=1}^{W}\sum_{h=1}^{H}||UI_{CI}{}^{c,w,h}-UI_{GCI}{}^{c,w,h}|{|}_{2}^{2}$$

Here, $UI_{GCI}$ is generated clean UAV image and $UI_{CI}$ represents the corresponding real clean UAV image.

## 3. Experiments

### 3.1. Experimental Setting

#### 3.1.1. Experimental Data

Because of the lack of UAV image data sets for denoising training and assessment, a new UAV image set for training and testing the proposed networks was built for our experiments. The UAV image training set was obtained by a CW-30 UAV in Guiyang city, Guizhou Province. The camera was a H5D-50 with the focal length of 50 mm; the size of entire image was 8176 × 6132 pixels and the flying height was 600–800 m. For the convenience of training, we cut entire images into small images using Photoshop; 400 images of 360 × 360 pixels were used as the clean training data and different levels of noise were added to these clean images to make them noisy images. The testing set includes two parts; one part consists of 40 pieces of UAV images and the other consists of 100 pieces of smaller UAV images, comprised of images of cars and trucks. The testing set was obtained from other mapping areas. One part of the testing set was collected with a CW-30 UAV in Yangjiang city, Guangdong Province. The camera was a SWDC5 with a focal length of 50 mm; the size of entire image is 8206 × 6078 pixels and the flying height is 600–800 m. The other test dataset was obtained using a CW-10 UAV in Wuhan city, Hubei Province. The camera was a ILCE-7R with a focal length of 28 mm; the size of entire image is 7360 × 4916 pixels and the flying height is 400–600 m.

#### 3.1.2. Prameters Setting and Model Details

The entire networks are trained on a Nvidia GRID M60-8Q (8G) GPU using the tensorflow framework, the number of training iterations is 160 k. Due to the limited memory of the computer, the batch size of our experiment was 1. We used Aadm as an optimization algorithm and set the learning rate at 0.9. In the training process, we set *x* = 0.5 × 10^−3^, *y* = 2 × 10^−6^ (in Equation (2)) by experimental experiences. All strides were 1 in the generative model G, while all other convolutions were composed of 3 × 3 sized kernels, except for the last convolution with a 1 × 1 sized kernel. In the discriminative model D, the first six layers were composed of 4 × 4 sized kernels with a stride of 2; the next layer was composed of 1 × 1 sized kernels with a stride 1 and the last two layers were composed of 3 × 3 sized kernels with a stride 1. In generative model G and discriminative model D, all padding modes padding edges of kernels.

### 3.2. Comparison and Qualitative Evaluation

We added synthesized noise to the testing images with three noise levels: 20, 35 and 55, and compare the proposed method with several state-of-the-art methods used for denoising of remote sensing images. These comparative results are presented in the following subsections.

#### 3.2.1. Comparion in the Traditional Way

**Figure 2:**
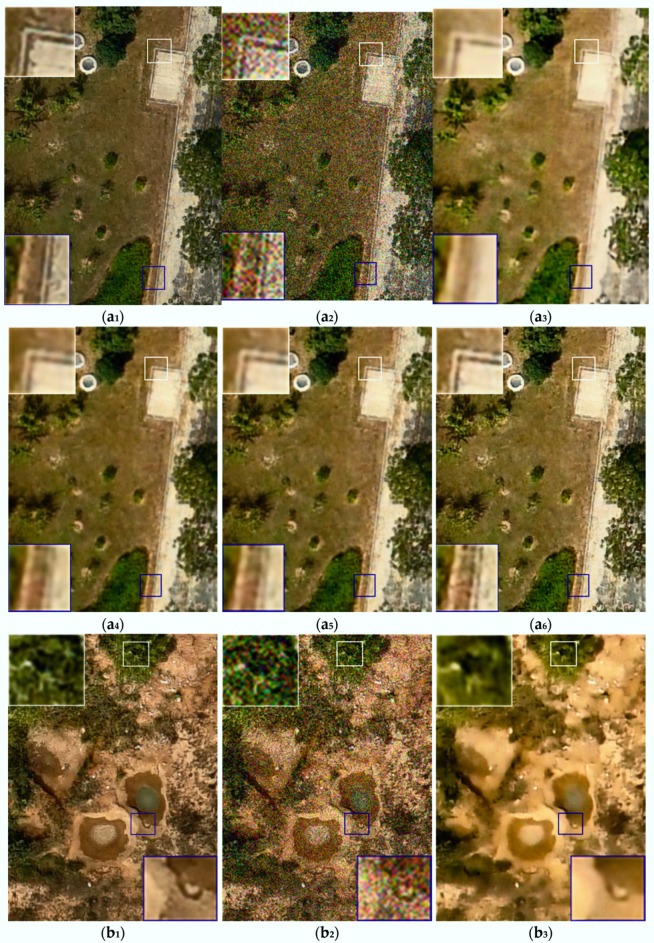

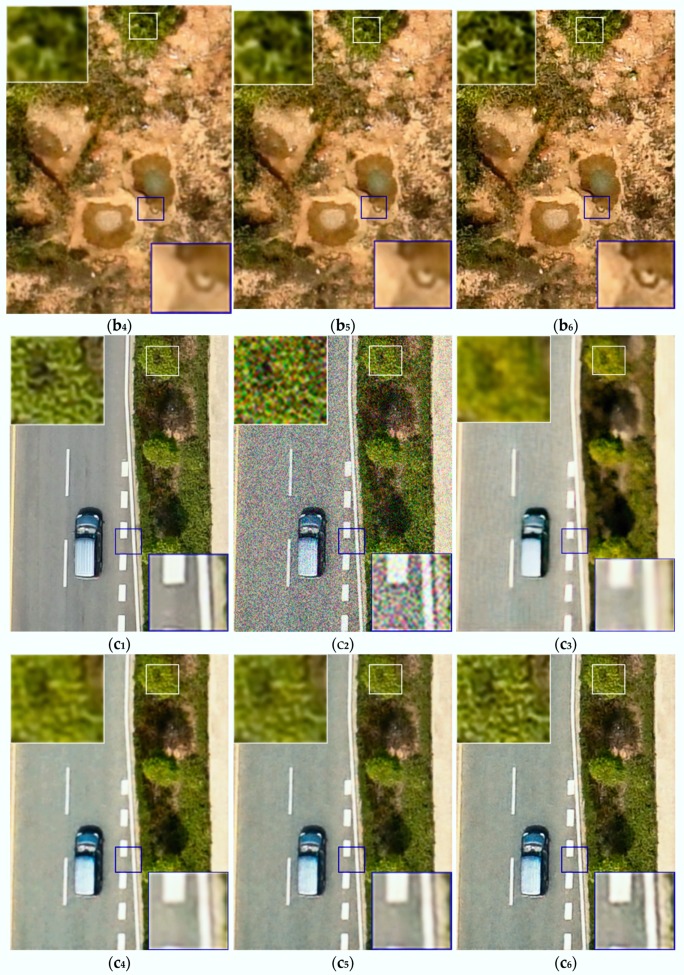

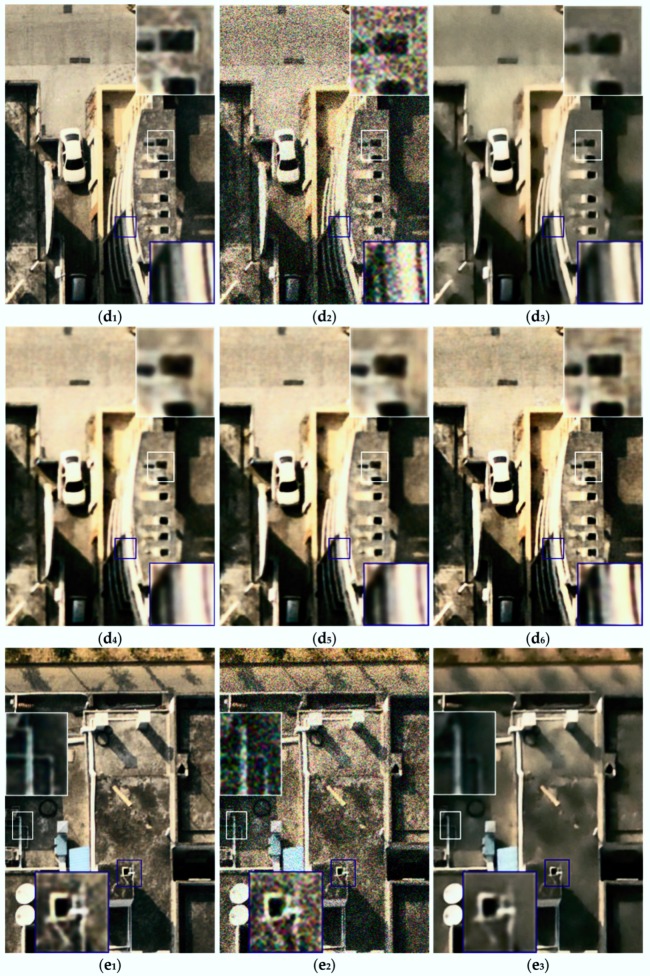

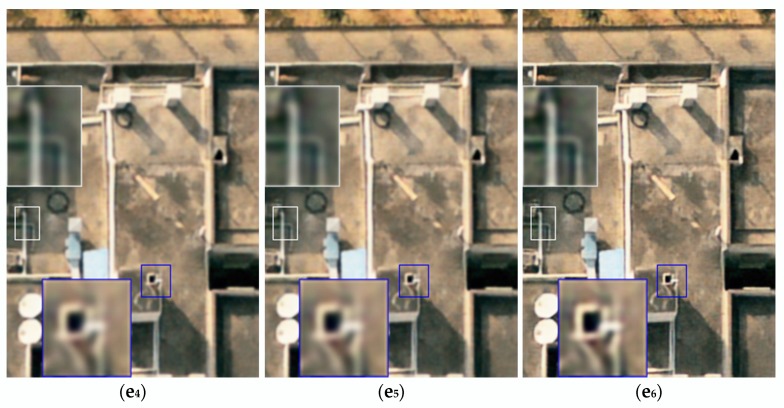
Testing results of several denoising methods for UAV images with several ground objects. In each group of images, the 1st image (**a_1_**,**b_1_**,**c_1_**,**d_1_**,**e_1_**) is the ground truth; the 2nd image (**a_2_**,**b_2_**,**c_2_**,**d_2_**,**e_2_**) is a noise image with noise level 35; and the 3rd image (**a_3_**,**b_3_**,**c_3_**,**d_3_**,**e_3_**) presents the denoising results of method [8]. The 4th image (**a_4_**,**b_4_**,**c_4_**,**d_4_**,**e_4_**) presents the denoising results of method [5]; the 5th image (**a_5_**,**b_5_**,**c_5_**,**d_5_**,**e_5_**) presents the denoising results of method [17]; and the 6th image (**a_6_**,**b_6_**,**c_6_**,**d_6_**,**e_6_**) presents the denoising results of the proposed method.

Figure 2 shows results when the noise level was 35. Images in columns a, b, c, d, and e are five randomly picked testing images showing different ground objects. The labels a_1_-e_1_ denotes the actual clean UAV images, the a_2_-e_2_ denotes the synthetic noise image, and the a_3_–e_3_ denotes the UAV image denoised by method [8]. The label a_4_–e_4_ denotes the denoised UAV image by method [5], the a_5_–e_5_ identifies the UAV image denoised by method [17], and the a_6_–e_6_ denotes the UAV image denoised by the proposed method. In each of the images, small rectangles in white and blue identify areas enlarged for examination in the larger white and blue rectangles in each series of images with different levels of noise.

**Figure 3:**
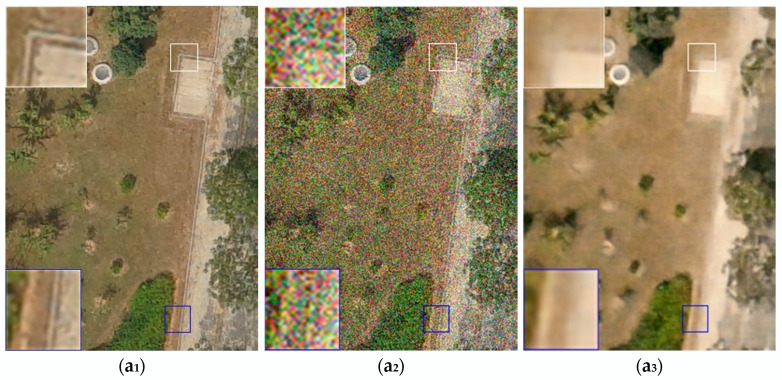

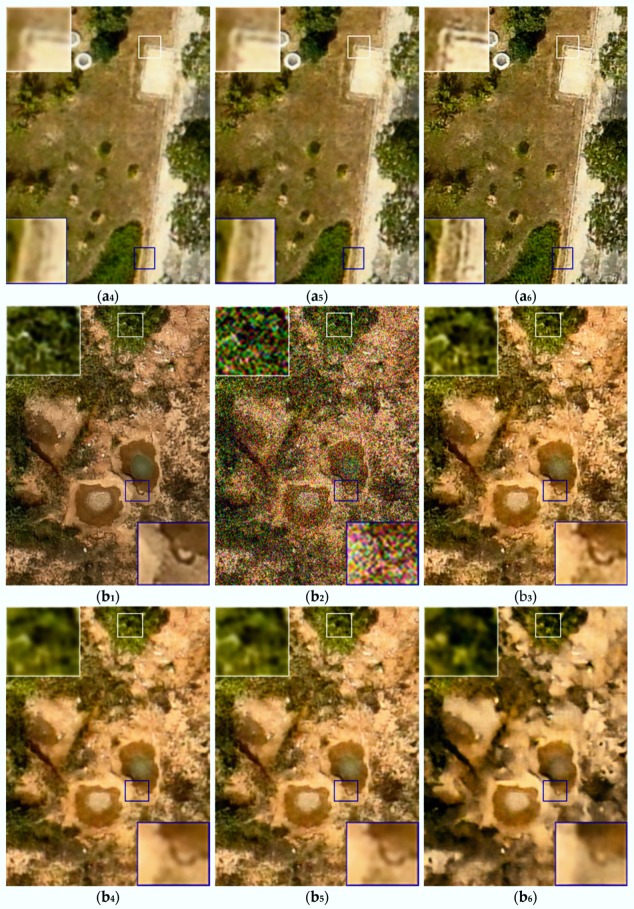

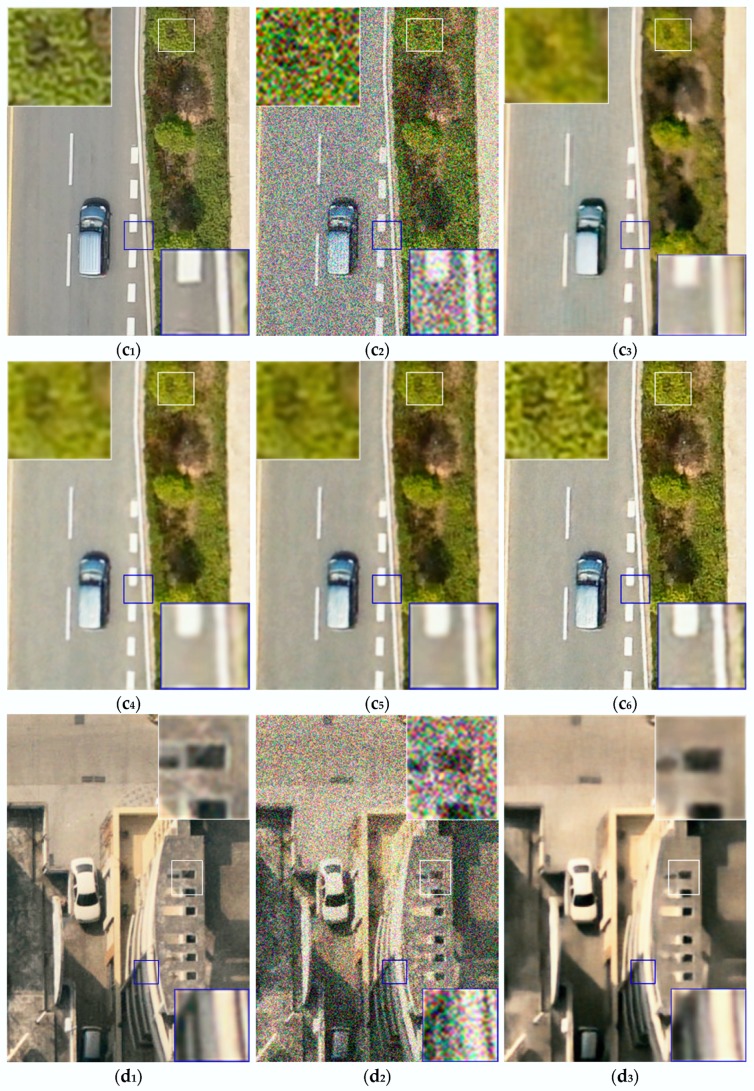

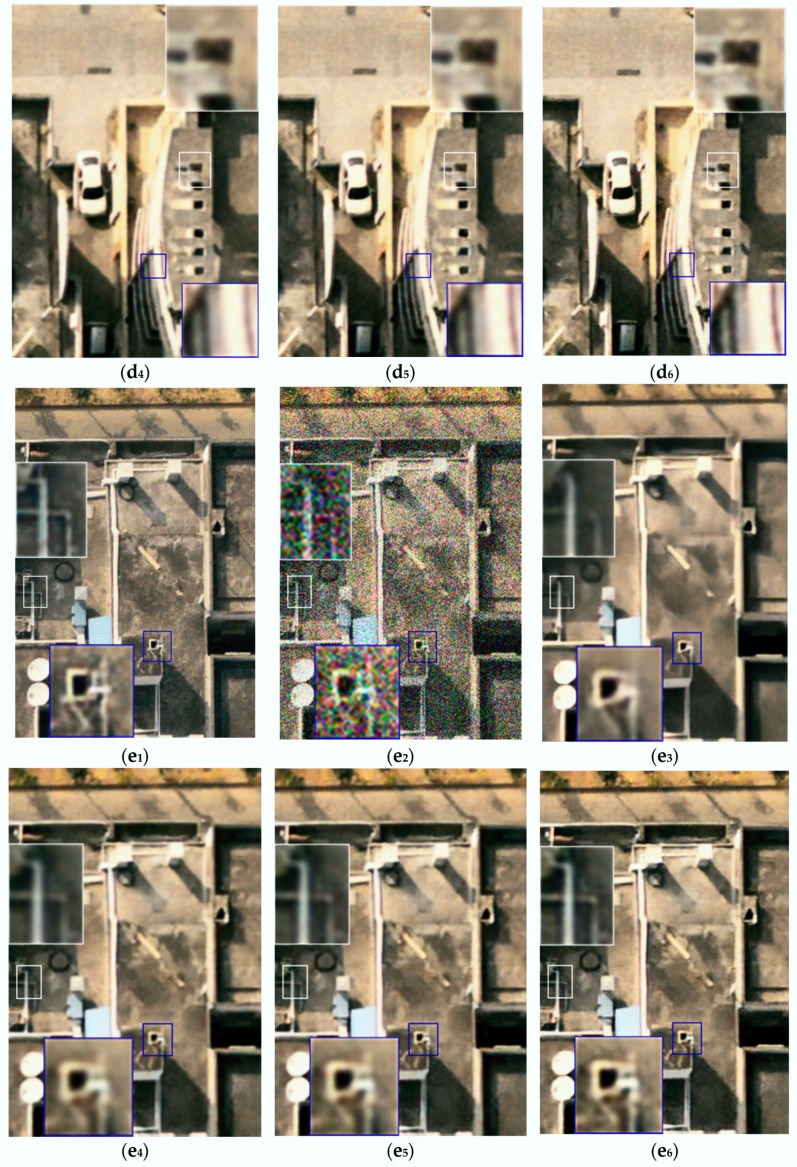
Testing results of several denoising methods for UAV images with several ground objects. In each group of images, the 1st image (**a_1_**,**b_1_**,**c_1_**,**d_1_**,**e_1_**)is the ground truth; the 2nd image (**a_2_**,**b_2_**,**c_2_**,**d_2_**,**e_2_**) is a noisy image with noise level 55; and the 3rd image (**a_3_**,**b_3_**,**c_3_**,**d_3_**,**e_3_**) presents the denoising results of method [8]. The 4th image (**a_4_**,**b_4_**,**c_4_**,**d_4_**,**e_4_**) presents the denoising results of method [5]; the 5th image (**a_5_**,**b_5_**,**c_5_**,**d_5_**,**e_5_**) presents the denoising results of method [17]; and the 6th image (**a_6_**,**b_6_**,**c_6_**,**d_6_**,**e_6_**) presents the denoising results of the proposed method.

In Figure 2 and Figure 3, the experimental results of five randomly picked testing images of different ground objects have been provided. In the testing experiments with different noise levels, it can be observed that the proposed method preserves more distinct ground edges and more clear ground objects textures. Meanwhile, the denoised UAV images by DUGAN are better in overall visual effect and closer to the true ground objects in terms of UAV image structures. Due to the fact that we have relatively deeper networks and the loss function can better preserve the overall styles of the UAV images, deeper ground feature information can be extracted, which is of great help for the later application of UAV images.

As can be seen in the images shown in Figure 2, at noise level 35, the proposed method outperforms the other tested methods. For example, in the e series of images, the full size and enlarged portion of the image processed using proposed method is clearer, with sharper edges than the results from the other tested methods. Figure 3 is similar to Figure 2, with the same labeling scheme, order, and enlarged details, but shows results at noise level 55. It can be seen in Figure 2 and Figure 3, a qualitative visual comparison of the proposed method and the other tested methods at different noise levels shows that the proposed method preserves distinct ground edges and clear ground object textures. The denoised UAV images produced by the proposed method are better in overall visual effect and closer to the true ground objects in terms of UAV image structures. Because the proposed model have relatively deeper networks and the loss function thus preserves the structure of terrain shown in the UAV images, so that deeper ground feature information can be extracted, which is of great help in later applications using these UAV images.

Table 1 and Table 2 quantitatively compare denoised images obtained using the tested denoising methods using the Peak Signal to Noise Ratio (PSNR) and Structural Similarity Index (SSIM). Table 1 presents comparative image denoising results using PNSR for the tested methods at different noise levels. The columns indicate the tested method, while rows 3–7 represent the PSNR values of denoised images obtained using the test denoising methods when the noise level is 20. The rows 10–14 represent the PSNR values of denoised images obtained with several denoising methods when the noise level is 35, rows 17–21 represent the PSNR values of denoised images obtained with several denoising methods when the noise level is 55. Table 2 is similar to Table 1, but shows results using SSIM for the tested methods at different noise levels.

As can be seen in Table 1 and Table 2, the proposed method has the highest PSNR and SSIM [37] in different noise levels can be observed, this is consistent with the visual effects of the denoised UAV images.

#### 3.2.2. Compare Denoising Results Using Image Matching

To verify our method further, the denoised UAV images gathered by several methods are matched with the real clean UAV images so that the results of the matching experiments were employed to compare the outcomes of denoising. Scale Invariant Feature Transform (SIFT) [38] is a well-known matching algorithm in image matching based on matching methods of image local features. The quality of the matching results can often reflect the similarities of the local characteristics of the two images, and the number of matching point pairs is the standard for judging the quality of matching results. In terms of the professional characteristics, the textures of ground features are important expressions of the UAV images of local features. Therefore, the matching results of denoising UAV images by SIFT can indicate the denoising results. 

**Figure 4:**
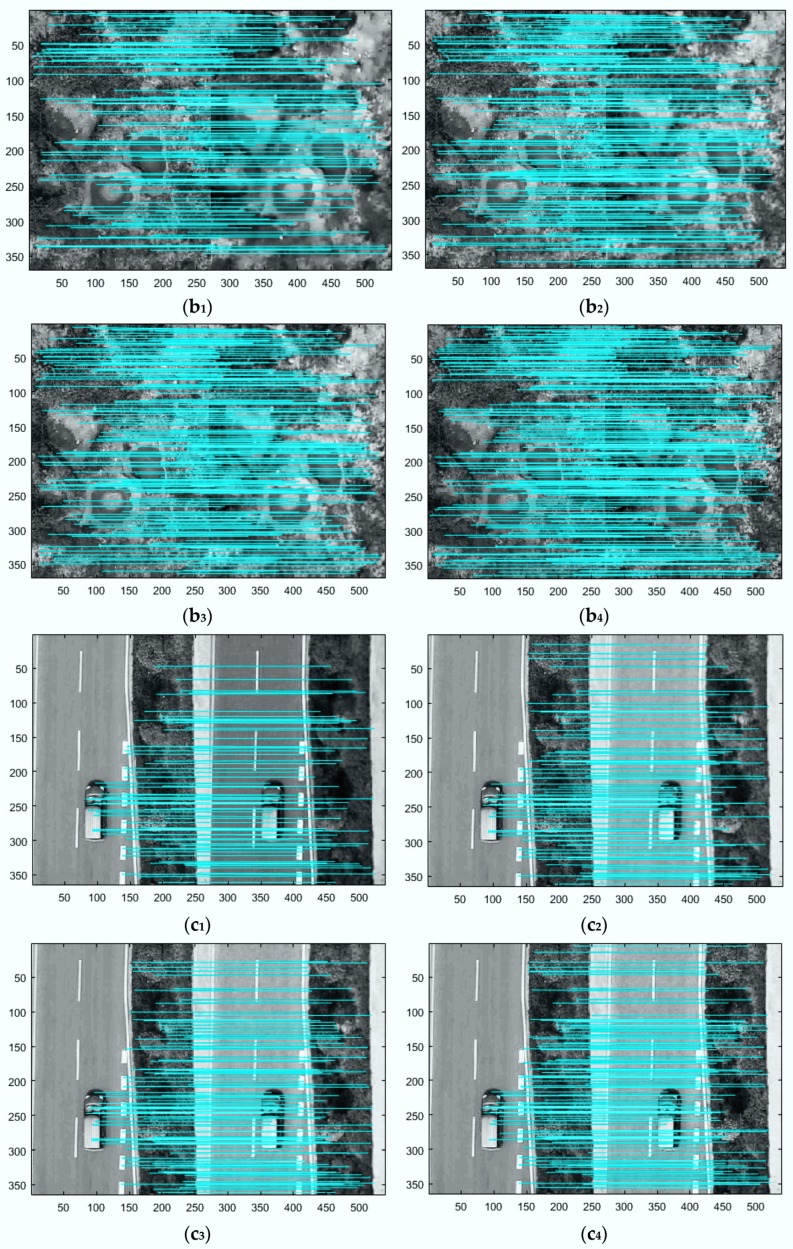

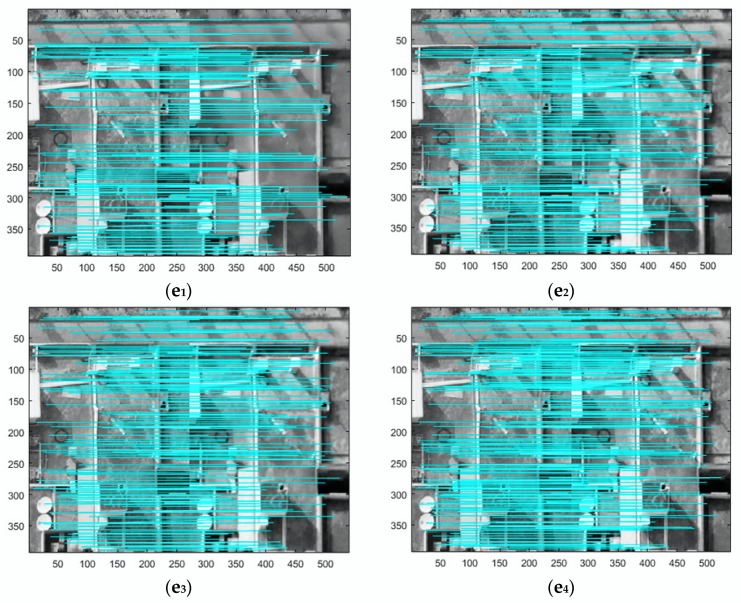
Due to the length concerns, we only show the matching results of 3 images (**b**,**c**,**e**). In each matching image, the left image is the original real clean UAV image, and the right image is the denoised UAV image (noise level 35). The 1st image (**b_1_**,**c_1_**,**e_1_**) presents matching results from SIFT and the denoised UAV images using method [8] and original real clean UAV images. The 2nd image (**b_2_**,**c_2_**,**e_2_**) presents matching results from SIFT and the denoised UAV images using method [5] and the original real clean UAV images. The 3rd image (**b_3_**,**c_3_**,**e_3_**) presents matching results from SIFT and the denoised UAV images created by method [17] and original real clean UAV images; and the 4th image (**b_4_**,**c_4_**,**e_4_**) presents matching results from SIFT and the denoised UAV images using proposed method and the original real clean UAV images.

**Figure 5:**
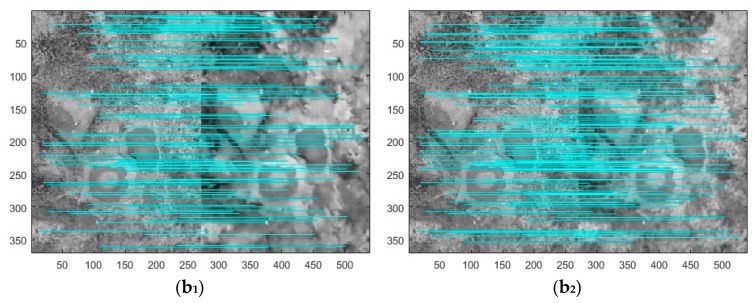

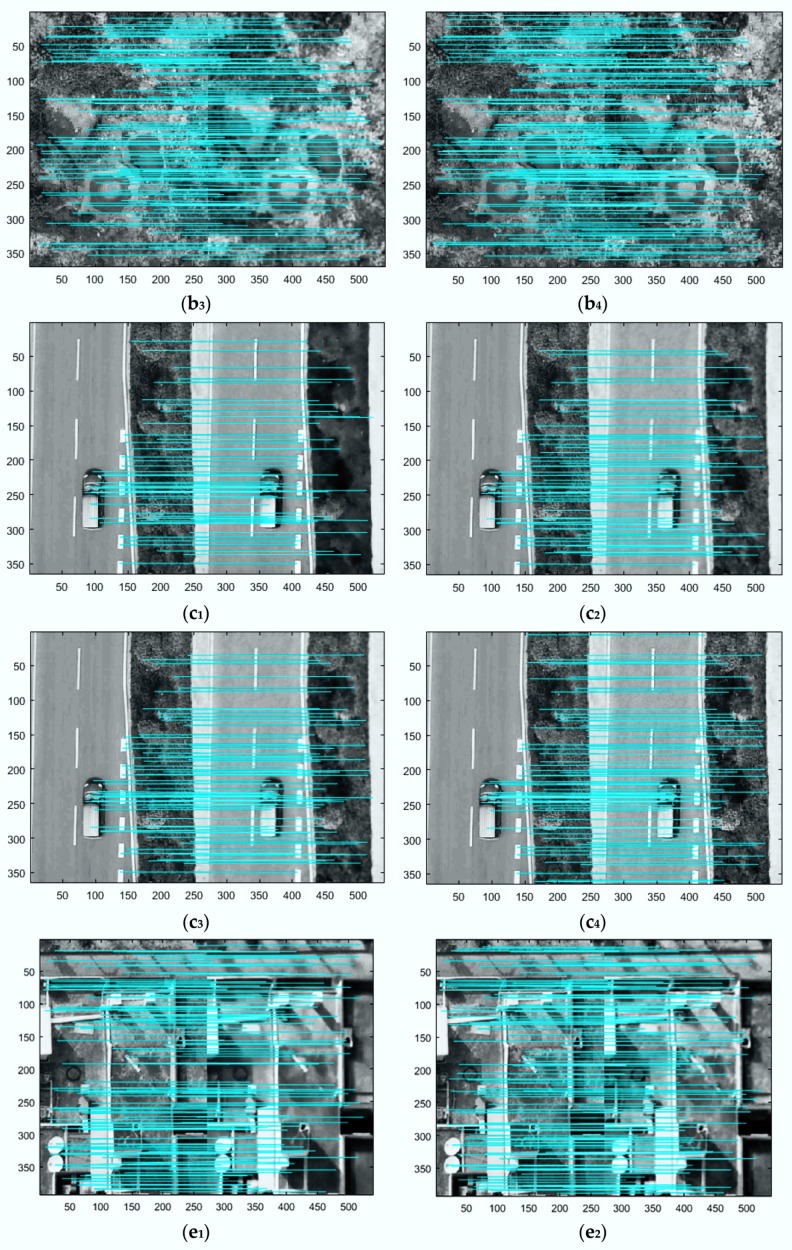

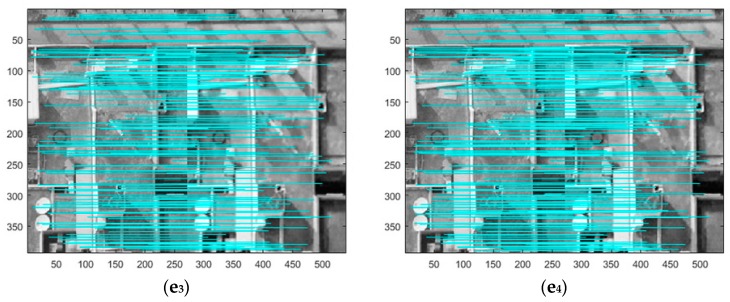
In each matching image, the left image is the original real clean UAV image, and the right image is the denoised UAV image (noise level 55). The 1st image (**b_1_**,**c_1_**,**e_1_**) presents matching results from SIFT and the denoised UAV images using method [8] and original real clean UAV images. The 2nd image (**b_2_**,**c_2_**,**e_2_**) presents matching results from SIFT and the denoised UAV images using method [5] and the original real clean UAV images. The 3rd image (**b_3_**,**c_3_**,**e_3_**) presents matching results from SIFT and the denoised UAV images created by method [17] and original real clean UAV images; and the 4th image (**b_4_**,**c_4_**,**e_4_**) presents matching results from SIFT and the denoised UAV images using proposed method and the original real clean UAV images.

In order to ensure the objectivity of the experiment, the denoising results of the above five pieces of UAV images are used to match with the corresponding real clean UAV images by SIFT algorithm. Figure 4 and Figure 5 shows the matching results by SIFT. In Figure 4, when the noise level is 35, the labels b_1_–e_1_ indicates the SIFT matching result of the original clean image and the denoised image obtained by method [8], the labels b_2_–e_2_ indicates the SIFT matching result of the original clean image and the denoised image obtained by method [5], the labels b_3_–e_3_ indicates the SIFT matching result of the original clean image and the denoised image obtained by method [17], the labels b_4_–e_4_ indicates the SIFT matching result of the original clean image and the denoised image obtained by the proposed method. Figure 5 is similar to Figure 4, but shows results at noise level 55. 

In Table 3, Column 1 denotes five randomly picked testing images, Column 2 denotes correct matching pairs of the original clean image and the denoised image obtained by method [8], Column 3 denotes correct matching pairs of the original clean image and the denoised image obtained by method [5], Column 4 denotes correct matching pairs of the original clean image and the denoised image obtained by method [17], Column 5 denotes correct matching pairs of the original clean image and the denoised image obtained by proposed method.

As is shown in Figure 3, Figure 4 and Figure 5, it can be observed that the denoised images generated by proposed method in two noise levels (noise level: 35, 55) obtain more correct matching pairs than other methods in number.

#### 3.2.3. Compare Denoising Results Using Image Classification

**Figure 6:**
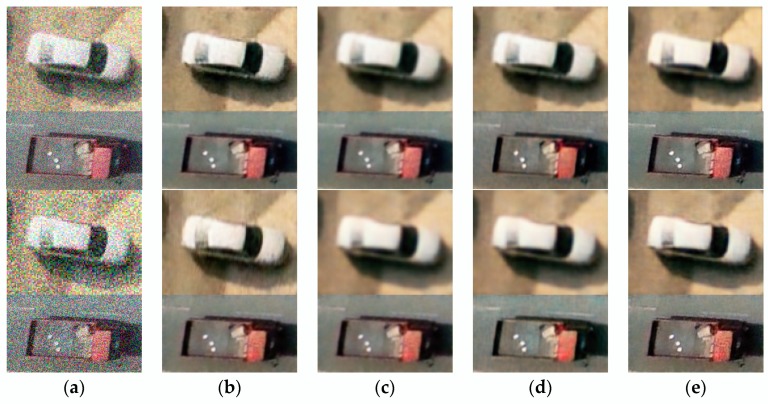
(**a**) Represent the noisy images of cars and trucks with different noise levels; (**b**) Represent the denoising results of method [8]; (**c**) Represent the denoising results of method [5]; (**d**) Represent the denoising results of method [17]; (**e**) Represent the denoising results of proposed method.

In this experiment, we used 100 denoised UAV images (60 cars and 40 trucks) to conduct images classification experiment to compare denoising results of several methods (Figure 6). The classification networks consist of five layers: one input layer, three hidden convolutional neural networks (convolution kernel size is 3), and a softmax output layer. We used 1 and 0 to represent clean images of cars and trucks, respectively. Then, we employed 80 clean UAV images to train classification networks and 20 clean UAV images to test the trained classification networks. After completing the classification networks training, the classification correct rate of the testing images reaches 85%. The definition of classification correct rate is shown in Equation (6):

(6) $$\mathrm{Correct}\;\mathrm{rate}=\frac{N_{C}}{N_{T}}$$

where, $N_{C}$ is the number of correctly classified UAV images and $N_{T}$ is the number of total UAV images. We add different noise levels (noise level: 35, 55) to those 100 clean images, and obtain denoised images by several denoising methods. The trained classification networks are used to classify the denoised UAV images. From Table 4, it can be seen that the classification results of denoised images obtained by our method enjoy a higher accuracy rate, which can reflect indirectly that denoised images obatined by our method are more similar to clean images.

Through these three comparative experiments with different noise levels, it can be observed that the denoised images obtained by the proposed method can obtain better denoising results. In the SIFT matching algorithm, the denoised images of our method gets more correct matching pairs, which shows that our method can better restore the local characteristics of UAV images and preserve the textures, as observed via classification experiments with denoised images that denoised images generated by our method are more similar to real clean images.

## 4. Conclusions

In this paper, we use perceptual reconstruction loss function and pixel-based loss function and propose a method of denoising UAV images based on generative adverserial networks. According to the special requirements of the UAV images for the textures of ground objects, multiple residual blocks are used to build a deep learning framework, which makes the denoised images obtain more details of the texture features. The denoising results of this method yield better results in the traditional evaluation methods. Meanwhile, in the experiments based on local feature matching and image classification, good results are achieved, which is helpful for subsequent applications of UAV images. In the deep networks, each layer of the networks can be viewed as a filter. The deeper networks have more filters of UAV images denoising, which make the entire networks become more nonlinear. The nonlinearity of the networks is the critical factor of the proposed method, which makes it superior to other denoising methods. In the future work, we will further explore how to simulate real UAV noise so as to obtain more ideal denosing effect of UAV images based on adversative learning.

## Figures and Tables

**Table 1:** Quantitative measurement results using PSNR (dB) on UAV testing images with different noise levels.

Images	Method [8]	Method [5]	Method [17]	Ours
	Noise Level: 20			
a	30.7543	30.9581	31.0174	31.2741
b	30.3965	30.5583	30.6967	30.9458
c	31.6907	31.9678	31.8534	32.1033
d	29.6358	29.8145	29.9543	30.2104
e	30.2235	30.4169	30.5576	30.7562
Average results ^1^	30.5942	30.7657	30.9856	31.1123
**Images**	**Noise Level: 35**			
a	28.8165	28.6967	28.8871	29.0564
b	28.3084	28.5758	28.4312	28.9687
c	29.4874	29.7321	29.7276	29.9576
d	27.5172	27.6954	27.8958	28.1354
e	28.2913	28.4782	28.6184	28.7858
Average results	28.4358	28.6127	28.7968	28.9854
**Images**	**Noise Level: 55**			
a	26.1869	26.3112	26.4984	26.7156
b	25.9276	26.1164	26.3658	26.5942
c	27.1962	27.3335	27.4795	27.6614
d	25.4956	25.6442	25.7841	25.9612
e	26.0517	26.2654	26.3127	26.5154
Average results	26.0014	26.2424	26.4912	26.6576

^1^ The average results of 40 UAV test images.

**Table 2:** Quantitative measurement results using SSIM on UAV testing images with different noise levels.

Images	Method [8]	Method [5]	Method [17]	Ours
	Noise Level: 20			
a	0.8799	0.8818	0.8841	0.8857
b	0.8776	0.8797	0.8804	0.8823
c	0.8857	0.8897	0.8895	0.8912
d	0.8704	0.8723	0.8741	0.8765
e	0.8731	0.8763	0.8779	0.8806
Average results	0.8756	0.8781	0.8796	0.8821
**Images**	**Noise Level: 35**			
a	0.8345	0.8362	0.8379	0.8406
b	0.8309	0.8339	0.8352	0.8371
c	0.8398	0.8441	0.8433	0.8468
d	0.8231	0.8249	0.8271	0.8289
e	0.8276	0.8303	0.8319	0.8343
Average results	0.8289	0.8312	0.8333	0.8369
**Images**	**Noise Level: 55**			
a	0.7682	0.7701	0.7719	0.7745
b	0.7639	0.7661	0.7679	0.7701
c	0.7739	0.7792	0.7798	0.7819
d	0.7245	0.7271	0.7289	0.7315
e	0.7589	0.7618	0.7641	0.7658
Average results	0.7601	0.7647	0.7662	0.7689

**Table 3:** The comparison of the correct matching pairs between denoised images and the corresponding clean UAV images obtained by SIFT.

Images	Method [8]	Method [5]	Method [17]	Ours
	Noise Level 35/55	Noise Level 35/55	Noise Level 35/55	Noise Level 35/55
a	110/53	114/74	127/78	150/88
b	160/101	205/141	240/145	274/154
c	96/66	104/70	118/76	135/78
d	136/115	138/129	157/144	192/154
e	177/154	192/162	211/179	260/215

**Table 4:** The comparison of the correct rate of denoised images classification.

Noise Level	Correct Rate of Method [8]	Correct Rate of Method [5]	Correct Rate of Method [17]	Correct Rate of Ours Method
35	67%	71%	73%	78%
55	62%	65%	70%	72%
